# Enhancing sanitation efficiency in red meat processing: a novel enzymatic approach

**DOI:** 10.3389/fmicb.2026.1818470

**Published:** 2026-04-21

**Authors:** Anna Macdonald, Argenis Rodas-Gonzalez, Tim McAllister, Xianqin Yang, Claudia Narvaez-Bravo

**Affiliations:** 1 Faculty of Agriculture and Food Science, University of Manitoba, Winnipeg, MB, Canada; 2 Lethbridge Research and Development Centre, Agriculture and AgriFood Canada, Lethbridge, AB, Canada; 3 Agriculture and AgriFood Canada, Lacombe, AB, Canada

**Keywords:** biofilm, *E. coli* O157:H7, enzymes, food safety, red meat, sanitation, sustainable

## Abstract

Residual organic matter on food contact surfaces (FCS) in meat processing plants promotes bacterial attachment and persistence, compromising meat safety and quality. Conventional hot-water cleaning (55–60 °C) can exacerbate this problem by heat-setting proteinaceous residues, leading to the formation of conditioning films that protect microorganisms from subsequent sanitation. A low-temperature, enzyme-based cleaning strategy was specifically designed to disrupt meat-derived residues before thermal fixation occurs, representing a departure from conventional heat-dependent cleaning regimes. A targeted enzyme screening approach was used to identify formulations active at reduced operating temperatures (40–50 °C) and neutral pH, conditions compatible with energy-efficient cleaning-in-place systems. A protease–lipase combination was selected and evaluated on stainless steel and conveyor belt materials contaminated with beef residues. Surface swabbing assays (ATP and protein) quantified the hygienic properties of surfaces post-treatment, showing that the enzyme treatment reduced the frequency of detection of protein on both surface types and reduced ATP levels by approximately 93% on FCS (*p* < 0.0001). To address microbial persistence beyond residue removal, the formulation was further optimized by incorporating cellulases and an amylase to target extracellular polymeric substances within *Escherichia coli* O157:H7 biofilms. The enzymatic cocktail reduced biofilm mass significantly (*p* < 0.0001). There was no statistical difference between negative control wells and enzymatic treatment wells (*p* = 0.3060), demonstrating near eradication of the biofilm matrix. Enzymatic cleaning regimes show great promise for sustainably enhancing food safety practices in meat processing plants, with lower operating temperatures and more mild solution pH.

## 1 Introduction

Despite the red meat industry’s significant contributions to North American diets and economies, meat producers face unresolved production challenges (USDA, 2024; Agriculture and Agri-Food Canada, 2025). Notably, red meat producers (referring to meat derived from cattle and swine) face building pressure from consumers and regulatory bodies to adopt more sustainable practices (De Boer et al., 2013). Going forward, the sector must actively pursue and implement innovative strategies to promote sustainable operations and minimize its environmental footprint. In particular, meat processing plants use sizable amounts of freshwater to maintain operating standards (Bustillo-Lecompte and Mehrvar, 2015), with recent estimates of approximately 11–16.5 liters of freshwater consumed during processing each kilogram of boneless beef in Canadian meat plants, depending on the plant (Legesse et al., 2018). Despite global efforts to preserve freshwater resources, strategies to reduce water consumption within meat processing plants are sparse (Bustillo-Lecompte and Mehrvar, 2015). Water-saving methods are needed to alleviate some of the ecological burden; however, current water usage is tied to pathogen reduction procedures vital for food safety, such as chemical carcass washes (Wu and Mittal, 2012). Standard sanitation operating procedures to clean and disinfect meat processing lines employ sequential water-intensive steps, comprising initial removal of meat residues, detergent cleaning, rinsing, and application of chemical disinfectants (Wang et al., 2018). These sanitation procedures cannot be eliminated without compromising food safety. Striking a balance is crucial; strategies to reduce water consumption must not compromise food safety practices.

From a food safety perspective, conventional sanitation regimes relying on high-pressure, hot water (60 °C) sprays can be detrimental to the hygienic quality of processing plants (Yadav et al., 2025). Hot water sprays repeatedly generate steams and aerosols, raising environmental humidity and fostering pervasive microbial growth (Lutgring et al., 1997). Moreover, the creation of contaminated aerosols may spread microorganisms to new surfaces, leading to the establishment of bacterial populations on otherwise clean surfaces (Mohammad et al., 2021). Additionally, the temperature of water applied may be counterproductive to cleaning efforts, as proteins originating from muscle tissues can aggregate at 55–60 °C, promoting hardening of proteinaceous residues (Tornberg, 2005).

Interestingly, enzymatic treatments have been effective in reducing organic residues and microbial biofilms, while minimizing water usage and reliance on harsh chemicals and temperatures (Allie et al., 2003; Timmerman et al., 2016; Puga et al., 2018; Guerrero-Navarro et al., 2019; Delhalle et al., 2020; Mnif et al., 2020; Iñiguez-Moreno et al., 2021; Rahman et al., 2022; Esposito and Turku, 2023; De Medici et al., 2024). The hygiene-enhancing properties of enzymes can be further optimized by combining them with surfactants to suspend hydrolyzed substrates in solution, preventing reabsorption onto food contact surfaces and facilitating effective rinsing (Allie et al., 2003; Timmerman et al., 2016). Despite their apparent benefits, studies exploring the use of enzymes to enhance sanitation procedures in meat processing plants are sparse. It is hypothesized that enzymatic treatments will reduce proteinaceous contamination and disrupt biofilms in the meat fabrication environment, while providing a milder alternative to strong caustic chemicals. Enzymatic treatments may pose great potential to assist meat producers in implementing targeted and sustainable sanitation solutions. The main objectives of this study were to (1) design and select enzymatic solutions that are comparatively more eco-friendly to conventional caustic chemical treatments and are effective under reduced operating temperatures (40–50 °C), (2) assess the ability of enzymatic treatments to reduce conditioning films following contamination with beef products, and (3) evaluate the efficacy of enzymatic treatments to reduce meat-associated pathogen biofilms.

## 2 Materials and methods

### 2.1 Materials

To specifically target meat-based residues, nine commercially sourced enzyme mixtures were screened in preliminary testing to select suitable candidates to target conditioning films. Four lipase mixtures were included, sourced from *Aspergillus oryzae, Aspergillus niger*, wheat germ, and *Pseudomonas cepacia*, in addition to five protease products sourced from *Aspergillus oryzae*, *Streptomyces griseus*, *Bacillus amyloliquefaciens,* bromelain (from pineapple), and papain (from papaya). To target the biofilm matrix, three additional enzymes were selected, including cellulases from *Trichoderma reesei* and *Aspergillus niger,* and an *α*-amylase from *Bacillus* sp. (Cat:10069). Enzymes were purchased from Sigma Aldrich (St. Louis, Missouri, United States), except for papain, purchased from Worthington Biochemical Corporation (Lakewood, New Jersey, United States). All enzymes were lyophilized, except for protease from *Bacillus amyloliquefaciens,* and lipase and protease from *Aspergillus oryzae*, which were liquid solutions. Enzymes were prepared 0.1% (w/v or v/v) in Butterfield’s Phosphate Buffer (pH 7.2), unless otherwise specified. Enzymes were measured in weight or volume rather than enzymatic units to consistently incorporate a variety of enzymes under a breadth of experimental conditions and assays (Mnif et al., 2020). After preparation, enzyme solutions were held at 4 °C for no longer than 14 days (ThermoScientific, 2009). Enzyme stability under these parameters was verified during enzyme activity testing. Chemical reagents and detergent components were purchased from Sigma Aldrich unless otherwise specified. Nitrophenyl palmitate and azocasein substrates were purchased from ThermoFisher Scientific (Waltham, MA, USA) and Megazyme Int. (Bray, Wicklow, Ireland), respectively. Thermoplastic polyurethane (TPU) and 304-stainless steel (SS) coupons were obtained from NuTech Conveyor Components (Milton, CA, United States) and Pegen Industries Inc., (Stittsville, CA, United States). The selected bacterial strain for biofilm formation was *Escherichia coli* O157:H7 R508, originated from bovine feces and was stored at −80 °C until use. Beef products used to contaminate surfaces were purchased from a local grocery store and stored at −20 °C until use.

### 2.2 Methods

#### 2.2.1 Enzymatic treatment of meat-based conditioning films

##### 2.2.1.1 Enzyme activity testing

Preliminary testing was performed to select lipases and proteases that remained active under conditions relevant to the study objectives, including reduced operating temperatures, neutral pH, and the presence of common cleaning compounds. The experimental parameters selected were based on those employed by other research groups developing sanitation regimes with reduced environmental impacts (Guerrero-Navarro et al., 2022). Enzymes demonstrating high activity under experimental conditions were additionally screened for retained activity when used in combination with other enzymes and various detergent components. The two lipases and two proteases which performed well in all preliminary experiments were selected for the treatment of contaminated food contact surfaces.

The lipolytic activity was assessed following procedures previously described (Allie et al., 2003; Vo et al., 2022). Briefly, the enzyme solutions were each mixed with nitrophenyl palmitate in suspension. Nitrophenyl palmitate is converted to 4-nitrophenol through enzymatic digestion which produces a measurable change in absorbance proportional to lipolytic activity. After 5 minutes incubation at either 40 °C or 50 °C, the amount of enzymatic hydrolysis of the substrate can be detected by a spectrophotometer (Genesys 20, ThermoScientific) at 410 nanometers.

The assay requires preparation of Solution 1: [225 mL Butterfield’s Phosphate Buffer (pH 7.2), 0.5-gram gum Arabic (Texturestar), and 2.0 grams Triton X-100 (Alfa-Aesar)] and Solution 2: [30 mL of 2-propanol and 90 milligrams of nitrophenyl palmitate (ThermoFisher)]. Prior to each trial, one part of Solution Two is slowly added to nine parts of Solution One while stirring rapidly at the chosen temperature of the assay. The combined Reaction Mixture was used promptly as the solution deteriorates rapidly, becoming cloudy and less accurate if not used within an hour. Each reaction tube contained 2.7 mL of freshly prepared Reaction Mixture held at the experimental temperature. 300 μL of each enzyme solution were aliquoted into the Reaction Mixture and incubated for 5 min. Absorbance was immediately measured by the spectrophotometer. Each enzyme reaction was compared against a reaction blank, containing only the Reaction Mixture and BPB, which served as the negative control. One unit of lipase activity was determined by measuring the increase in absorbance of *para-*nitrophenol as nitrophenol palmitate is hydrolyzed, according to Allie et al. (2003), based on the Beer–Lambert law (Supplementary Table 1).

The proteolytic activity of the enzymes was assessed by their ability to digest azocasein substrate (Boyce et al., 2010; Côlho et al., 2016). Azocasein was prepared in Tris–HCl buffer (8.0) according to formulation published by Megazyme International (Bray, Wicklow, Ireland). 125 μL of enzyme solution and azocasein were combined in microcentrifuge tubes and incubated for 30 min at 40 °C or 50 °C. After the 30 min elapsed, 750 μL of 10% trichloroacetic acid (Ricca, Arlington, TX, USA) was added to each reaction tube to terminate the reaction. The tubes were allowed to stand for 2 minutes, before being centrifuged (Eppendorf, Centrifuge 5,430) at 7000 x *g* for 5 minutes. 100 μL aliquots of supernatant were added to a 96-well, flat-bottomed microplate (Costar, Fisher Scientific) containing 100 μL of 1 M NaOH (Mallinkrodt) to neutralize the reaction solution. The plate was immediately read at 450 nanometer in a microplate reader (BioTek ELx800; BioTek Instruments Inc., Winooski, VT, United States). A reaction blank containing BPB instead of enzyme solution served as a negative control for baseline absorbance. Enzymatic activity was calculated according to Boyce et al. (2010). One unit of protease activity was defined as the amount of enzyme needed to produce an absorbance change of 1.0 under assay conditions and was adjusted to account for performing the assay in a microplate rather than a cuvette (Supplementary Table 1).

##### 2.2.1.2 Enzyme combination testing

After testing enzymatic activity individually, two lipases and two proteases were selected based on their strong activity under experimental conditions. The enzymes were then combined in a factorial approach (Table 1), along with all four enzymes in a final preparation referred to as Combination 6. Enzyme concentration was maintained at 0.1% in BPB. Protease and lipase activity were assessed using the previously described assays at 40 °C and 50 °C for each combination of enzymes. The two-lipase combination was only tested for lipase activity, and the two-protease combination was only tested for protease activity.

**Table 1:** Factorial approach to paired enzyme testing.

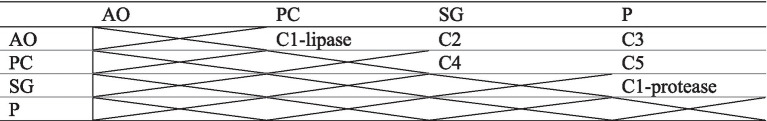

AO, Aspergillus oryzae lipase; PC, Pseudomonas cepacia lipase; SG, Streptomyces griseus protease; P, papain.

##### 2.2.1.3 Combination testing with detergent components

Detergent formulations commonly include a variety of chemical components, including surfactants, builders, and additives (U.S. Environmental Protection Agency, 1993). When used in combination, detergent components facilitate efficient solubilization of organic debris. Surfactants serve to remove dirt from surfaces, whereas builders increase the efficacy of surfactants and optimize their performance. Ammonium lauryl sulfate (ALS) (Sigma-Aldrich), sodium lauryl sulfate (SLS), and poloxamer-407 (POL) (Spectrum Chemical MFG Corp, New Brunswick, NJ, USA) are surfactants regularly found in cleaning formulations and were selected for inclusion in testing. Additionally, sodium borate (BOR) (Fisher Scientific, Ottawa, Ontario, Canada) is a builder commonly used in detergent formulations and was also included. The detergent components were prepared in BPB buffer (0.5%, w/v final concentration) and combined with enzyme solutions to promote optimal cleaning formulation efficacy.

The enzymatic activity of the selected enzyme combinations in the presence of detergent components was assessed using the previously described assays at 40 °C. Retained activity was calculated by dividing the activity with the chemical by the activity without the chemical (Arabacı and Karaytuğ, 2023). Enzymes which demonstrated the strongest proteolytic and lipolytic activity in the presence of chemical reagents were selected to include in treatment of contaminated coupons.

##### 2.2.1.4 Conditioning film generation

A combination of ground beef and beef exudate was used to contaminate FCS to recreate exposure conditions which may be found in meat processing facilities (Pang and Yuk, 2018; Nan et al., 2022). Beef exudate was produced by homogenizing ground beef with distilled water in a 1:2 ratio in a stomacher, before placing in a 4 °C refrigerator overnight. After 24 h, the solution was diluted to reach 1:6 beef to water. The liquid was filtered through cheesecloth and stored in a − 20 °C freezer for further use.

Thermoplastic polyurethane (TPU), commonly used for conveyor belts, and 304-grade stainless steel (SS) were selected to represent FCS in the beef processing facilities. Coupons used for microscopy were 2x2cm, whereas swabbing assays used 5x5cm coupons to provide a larger contact area. Before contaminating the surfaces, the coupons were thoroughly cleaned, according to Nan et al. (2022). The method was modified to remove sterilization, as complete sterilization is not common practice in industrial settings.

Larger coupons received 5 grams of ground beef, whereas smaller coupons used for microscopy received 2.5 grams of ground beef. Beef was pressed manually downwards onto the surface to ensure uniform deposition. Five mL of liquid beef purge was added per coupon to mimic exudative juices found on the meat processing line. A layer of parchment paper was applied to the meat, before 50-gram weights were applied to each contaminated coupon to simulate the pressure applied by a large piece of meat as previously described (Flores et al., 2006). The contaminated coupons were placed in a refrigerated environment (10 °C) for 16 h. In meat processing plants, it is common for two, 8-h fabrication shifts to occur before the overnight sanitation shift takes place. Therefore, 16 h represents the time surfaces may be exposed to food residues before thorough sanitation is performed. 10 °C was selected to represent the average temperature of a meat processing plant, which can fluctuate from 4 °C to 15 °C (Yang et al., 2017).

After 16 h had elapsed, the beef was removed, and surfaces were washed thrice with 10 mL of distilled water (40 °C). Surfaces were then treated with 4 mL (enough to fully cover the coupon) of one of the following treatments: conditioning film enzyme solution (CFES) (0.05% *Aspergillus oryzae* lipase, *Streptomyces griseus* protease, papain, 0.025% of *Pseudomonas cepacia* lipase, 0.5% SLS, 0.5% BOR in BPB), 2.5% PowerFoam (Epsilon Chemical), a detergent component control (0.5% SLS, 0.5% BOR), an enzyme control (CFES prepared without SLS/BOR), hot water or were not treated and served as a positive control. PowerFoam Plus is a strong chlorine-based cleaning and degreasing solution applied in past pilot-projects in Canadian meat processing facilities (Wang et al., 2018). The CFES, enzyme control, and detergent component control were applied at 40 °C, whereas PowerFoam Plus and hot water were applied at 60 °C. After 5 min of contact time, surfaces, including controls, were washed thrice with 10 mL of distilled water (40 °C) by flow using a serological pipette. Smaller coupons destined for imaging were separated and reserved at 4 °C.

##### 2.2.1.5 Food contact surface swabbing

ATP Clean Trace and Surface Protein Plus (Neogen, Lansing, Michigan, United States) swabs were selected to assess the remaining organic residue and protein following sanitation regime treatments. These methods were chosen as they are widely employed by the food industry for routine monitoring of cleaning and sanitation efficacy and allow the findings of this study to be readily translated into existing meat processing sanitation practices (Koti et al., 2026). Moreover, these methods provide a relatively high degree of sensitivity, allowing us to easily discern quantities of organic material on coupons following experimental treatments (Turner et al., 2010; Neogen, 2026). 5×5 cm coupons, post-contamination and treatment, were swabbed for 10 s in one direction, followed by a 90° turn and swabbed for another 10 s in the other direction, all while rotating the swab laterally to coat the test swab uniformly. ATP swabs were read in a Neogen Luminometer after 10 s. Protein Plus Swabs were allowed to sit for 10 min before assessing the degree of protein contamination. ATP readings are expressed as relative light units (RLU), whereas protein swabs yielded a semi-quantitative color change. The level of contamination was categorized according to color change (green: no contamination; gray-green: light contamination; light purple: moderate contamination; dark purple: heavy contamination). Surfaces serving as positive controls included SS and TPU coupons which underwent 16-h beef contamination and a warm water rinse. Surfaces serving as negative controls were exposed to distilled water for 16 h.

##### 2.2.1.6 Scanning electron microscopy

Imaging was conducted at the Manitoba Institute of Materials. Organic residues were fixed on TPU and SS coupons with 10% neutral buffered formalin (Sigma Aldrich) for 2 hours, before a 30-min wash in BPB, as described by Nan et al. (2022). Coupons were then dried overnight in a biosafety cabinet. TPU surfaces were coated with Gold–Palladium in a high vacuum mode (Denton Vacuum Desk II, Moorestown, NJ, USA). Surfaces were then observed on the scanning electron microscope on high vacuum mode at 10KV.

#### 2.2.2 Enzymatic disruption of biofilm matrix

##### 2.2.2.1 Selection of bacterial culture

To assess whether enzymes can additionally target biofilms which form in meat processing plants, biofilms were experimentally grown and treated with enzyme mixtures. Shiga-toxigenic *Escherichia coli* O157:H7 strain R508 was selected as a representative strong biofilm former and meat-associated pathogen (Nan et al., 2022). Single-species biofilms were grown in 96-well microplates (Costar, Fisher Scientific) to provide a biofilm matrix for treatment with enzyme mixtures. Reduction of mean biofilm density was quantified with the crystal violet staining technique following treatment with enzymatic solutions (Adator et al., 2018).

Frozen stock culture was streaked onto MacConkey agar plates (Hardy Diagnostics Inc., Santa Maria, CA, United States). A single isolated colony was selected and grown in Luria broth, no salt (LB-NS; Tryptone 10 g/L and yeast extract 5 g/L) to 10^8^ CFU/mL overnight at 25 °C. The culture was combined in 1:1 ratio with glycerol and stored in −80 °C for future experiments.

Curli and cellulose are compounds expressed by bacteria that are known to facilitate cell adhesion and biofilm formation (Saldaña et al., 2009). Phenotypic expression of curli and cellulose can be observed in the laboratory setting by streaking cultures onto Congo Red Agar plates or Luria-Bertani agar supplemented with 200 mg/L calcofluor dye (Sigma Aldrich), respectively, as described by Nan et al. (2022). To test expression, *E. coli* O157:H7 R508 culture was grown overnight at 25 °C to 10^8^ CFU/mL and streaked the following day onto the described agar plates before incubating at 37 °C for 24 h. Curli expression displayed on the Congo red plates as rough, red colonies, whereas curli-negative strains appear smooth and pale. Cellulose expression is determined by observing colonies under 366 nm ultraviolet light. Colonies positive for cellulose fluoresce, whereas colonies negative for cellulose expression do not. The experiment was performed in duplicate and repeated thrice.

##### 2.2.2.2 Biofilm formation, treatment, and enzymatic solution optimization

*Escherichia coli* O157:H7 R508 biofilms were grown in 96-well microplates. The culture was grown overnight in LB-NS at 25 °C to a concentration of 10^8^ CFU/mL. Preliminary biofilm trials used the overnight culture diluted 1:100 in LB-NS and grown for 5 days at 25 °C. After initial testing trials, the methods were revised to include beef exudate as a nutritional supplement (10% v/v) in the LB-NS. These adjustments were made to more closely replicate the biofilm composition of a meat processing plant. Additionally, the biofilms were allowed to develop for 6 days, as it was observed that the optical density was significantly higher after the additional day and would provide a more substantive biofilm to test the solutions on.

Once biofilms were established and ready to be treated, the inoculum was aspirated from each well. The biofilms were then washed with 300 μL of BPB three times to remove planktonic bacteria and allowed to dry for 10 min (Nan et al., 2022). In the first series of biofilm experiments, biofilms were treated with the same solutions applied to the FCS coupons (Supplementary Figure 1). Powerfoam Plus (2.5%) was applied to wells for 5 minutes at 60 °C following manufacturer recommendations, whereas enzyme solutions and detergent component controls were applied for 10 min at a constant temperature of 40 °C. Microplates were incubated to allow for optimal enzymatic digestion to occur (Lequette et al., 2010). However, incubation time was limited to 10 min to provide a timeframe that can be realistically implemented into sanitation regimes. After application, treatments were aspirated, and wells were washed thrice with 200 μL of warm, distilled water. Plates were then allowed to air dry for 10 min in the biological safety cabinet before staining with 0.1% (v/v) crystal violet (Nan et al., 2022). The absorbance was read using a microplate reader at 630 nm and analyzed using Gen5 software (BioTek ELx800; BioTek Instruments Inc., Winooski, VT, United States).

After completing pre-testing trials using treatments from the conditioning film experiments and assessing the data, the enzymatic treatments were adjusted to include cellulases and amylases to specifically target the biofilm matrix. The decision to adopt an enzymatic cocktail was made following a similar strategy employed by Mnif et al. (2020). Additionally, two concentrations and detergent components were tested to optimize the enzymatic solution (Table 2).

**Table 2:** Composition of solutions applied to *Escherichia coli O157:H7* R508 single species biofilms were grown in microplates for 6 days at 25 °C.

Active compound	Concentrations applied	Time, temperature applied
PowerFoam Plus	2.5% (v/v)	5 min, 60 °C
Detergent component control (either SLS or BOR)	0.5% (v/v)	10 min, 60 °C
Enzyme cocktail* in SLS/BOR (0.25% (w/v) sodium borate and 0.25% (w/v) sodium lauryl sulfate)	High concentration: 0.05% (w/v) of each enzyme Low concentration: 0.025% (w/v) of each enzyme	10 min, 40 °C
Enzyme cocktail* in SLS (0.5% (w/v) sodium lauryl sulfate)	High concentration: 0.05% (w/v) of each enzyme Low concentration: 0.025% (w/v) of each enzyme	10 min, 40 °C
Individual enzyme control (PC, SG, CTR, CAN, or AMY) in SLS (0.5% (w/v) sodium lauryl sulfate) OR SLS/BOR (0.25% (w/v) sodium borate and 0.25% (w/v) sodium lauryl sulfate)	High concentration: 0.05% (w/v) Low concentration: 0.025% (w/v)	10 min, 40 °C
Positive control	No treatment	-

^*^Enzyme cocktail contained protease from *Streptomyces griseus*, lipase from *Pseudomonas cepacia*, cellulase from *Aspergillus niger* and *Trichoderma reesei*, and α-amylase from *Bacillus* sp.

PC, *Pseudomonas cepacia* lipase; SG, *Streptomyces griseus* protease; CTR, cellulase from Trichoderma reesei; CAN, cellulase from *Aspergillus niger*; AMY, α-amylase from *Bacillus* sp.; SLS, sodium lauryl sulfate; BOR, sodium borate.

#### 2.2.3 Statistical analysis

All experiments were performed three times and in triplicate. Statistical analyses were performed using Statistical Analysis System Software (Cary, NC, United States) (SAS, 2012). Enzymatic activity assays and ATP swabbing experiments were analyzed by conducting a least means squares separation using a two-way Analysis of Variance (ANOVA) model. Preliminary biofilm reduction comparisons were conducted with a one-way ANOVA model. The biofilm treatment optimization assay was conducted with a two-way ANOVA model. Protein swabbing assays were categorically assessed with a Chi-Square distribution test. Significance levels were each set at *p* = 0.05.

## 3 Results

### 3.1 Protease and lipase testing

Nine enzyme mixtures were tested for their activity at 40 °C and 50 °C in proteolytic and lipolytic activity assays. Enzyme mixtures with higher activity showed higher absorbance on the spectrophotometer (Supplementary Table 1). In both reaction temperatures, *Pseudomonas cepacia* (*p* < 0.0001) and *Aspergillus oryzae* lipases (*p* < 0.0001) were the most active lipases, respectively. The proteases which demonstrated the highest activity in the azocasein assay at both reaction temperatures were from *Streptomyces griseus* (*p* < 0.0001) and papain (*p* < 0.0001), respectively.

### 3.2 Combined enzyme testing

The two most active enzyme solutions from both the lipase and protease groups were combined and tested for their lipolytic and proteolytic activity at 40 °C and 50 °C. For protease testing, combination 2 (lipase from *Aspergillus oryzae* + protease from *Streptomyces griseus*) and combination 4 (lipase from *Pseudomonas cepacia* + protease from *Streptomyces griseus*) demonstrated the highest activity among the solutions at 40 °C (OD = 2.47, 2.46, respectively). Similarly, at 50 °C, combinations 2, 4, and 6 (lipase from *Aspergillus oryzae* and *Pseudomonas cepacia,* and protease from *Streptomyces griseus* and papain) demonstrated the highest amount of protease activity (OD = 2.49, 2.52, 2.38). The highest lipase activity was demonstrated by combination 4, 5, (lipase from *Pseudomonas cepacia* + papain) and 6 at 40 °C (OD = 1.17, 1.30, 1.28, respectively), and by combination 5 and 6 at 50 °C (OD = 1.87, 1.73, respectively). After the conclusion of combined enzyme testing, combinations 4 and 6 were selected owing to their proteolytic and lipolytic action, and stability at both temperature levels, to be tested for their compatibility with conventional cleaning compounds.

### 3.3 Detergent component compatibility testing

**Figure 1:**
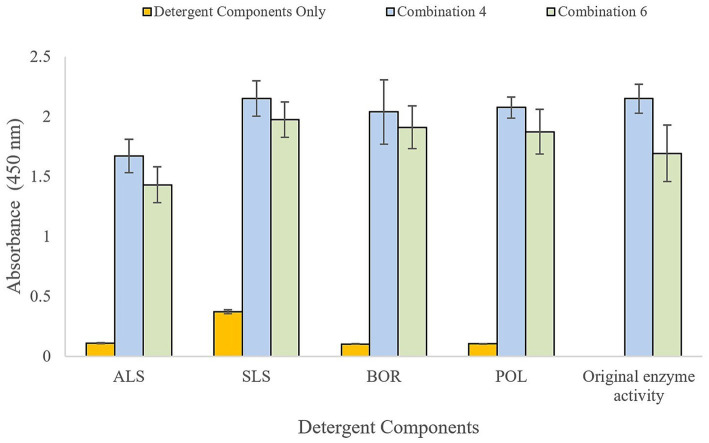
Protease testing of enzyme combination 4 (*Pseudomonas cepacia* and *Streptomyces griseus*) and 6 (*Aspergillus oryzae*, *Pseudomonas cepacia*, Papain and *Streptomyces griseus*) in presence of detergent components: ALS (ammonium lauryl sulfate), SLS (sodium lauryl sulfate), BOR (sodium borate), and POL (poloxamer 407). Change in absorbance proportional to azocasein substrate enzymatically digested. Total enzyme concentration 0.1% (w/v) and 0.5% detergent components in BPB.

**Figure 2:**
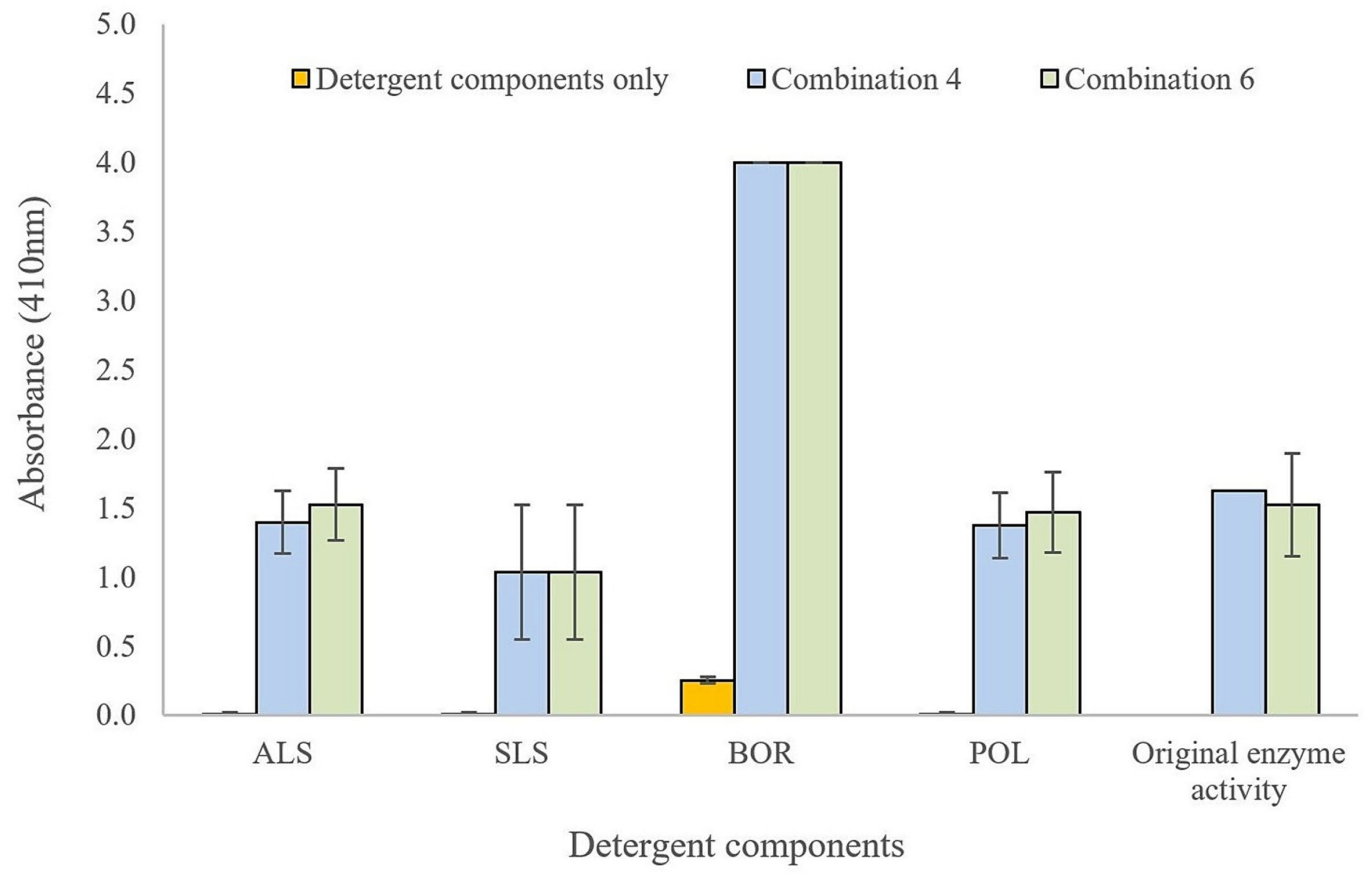
Lipase testing of enzyme combination 4 (*Pseudomonas cepacia* and *Streptomyces griseus*) and 6 (*Aspergillus oryzae*, *Pseudomonas cepacian*, Papain, and *Streptomyces griseus*) in presence of detergent components: ALS (ammonium lauryl sulfate), SLS (sodium lauryl sulfate), BOR (sodium borate), and POL (poloxamer 407). Change in absorbance proportional to nitrophenyl palmitate substrate enzymatically digested. Total enzyme concentration 0.1% (w/v) and 0.5% detergent components in BPB.

Enzyme combinations 4 and 6 demonstrated stability and retained activity in the presence of common cleaning reagents, namely ammonium lauryl sulfate (ALS), sodium lauryl sulfate (SLS), sodium borate (BOR), poloxamer 407 (POL) (Figures 1, 2). A synergistic relationship was observed between the proteases and sodium lauryl sulfate, and for the lipases and sodium borate. When including SLS in protease testing, combination 4 demonstrated 100% of retained original activity, whereas combination 6 had 117% of its original activity. When assessing lipase activity, BOR was able to boost the lipolytic activity from OD = 1.17 (combination 4) and OD = 1.28 (combination 6) to the maximum readable value on the spectrophotometer (OD = 4.00) for both combinations. Owing to the synergistic effects on enzyme activity, 0.5% sodium lauryl sulfate and 0.5% sodium borate were included in the final enzymatic formulation for testing experimentally contaminated FCS, referred to as conditioning film enzymatic solution (CFES). Combination 6, along with SLS and BOR, was selected for application to FCS, as previous research has found that more diverse combinations of enzymes are more effective as intervention treatments (Tsiaprazi-Stamou et al., 2019).

### 3.4 Assessment of sanitation effectiveness with conditioning film enzymatic solution (CFES)

Following treatment with conditioning film enzymatic solution, the coupons were swabbed with Protein Plus swabs to categorize the remaining level of proteinaceous contamination and statistically assessed using a Chi-square distribution (Tables 3, 4). The majority of CFES-treated SS coupons were categorized as “clean” (66.7%), compared to untreated coupons, which were “moderately” (44.4%) or “heavily” contaminated (55.6%) (Table 3). TPU coupons treated with CFES demonstrated slightly higher levels of contamination, with 44.4% classified as “clean” and 55.6% showing “light contamination.” The treatment which provided the highest frequency of “clean” surfaces following sanitation treatment was the PowerFoam Plus treatment (88.9% for SS, 77.8% for TPU). However, the detergent component control, containing SLS and BOR, had similar efficacy in cleaning the SS surfaces (88.9%). Overall, TPU showed higher retention of protein-based contamination compared to the SS surfaces.

**Table 3:** Distribution of protein contamination level following experimental treatments on SS surface.

Experimental treatment	Clean *n* (%)	Light *n* (%)	Moderate *n* (%)	Heavy *n* (%)
Conditioning film enzymatic solution	6 (66.7)	2 (33.3)	0 (0.0)	0 (0.0)
Enzyme control	1 (11.1)	7 (77.8)	1 (11.1)	0 (0.00)
Detergent component control	8 (88.9)	1 (11.1)	0 (0.0)	0 (0.0)
PowerFoam Plus	8 (88.9)	1 (11.1)	0 (0.0)	0 (0.0)
Hot water	4 (44.4)	5 (55.6)	0 (0.0)	0 (0.0)
Positive control	0 (0.0)	0 (0.0)	4 (44.4)	5 (55.6)
Negative control	9 (100.0)	0 (0.0)	0 (0.0)	0 (0.0)

Protein contamination was assessed categorically using Surface Protein Plus swabs (Neogen, Lansing, Michigan, United States) on stainless steel (SS) and thermoplastic polyurethane (TPU). Comparison of surface cleanliness frequency following experimental treatment with Chi-square analysis (*p* < 0.001).

**Table 4:** Distribution of contamination level following experimental treatments on TPU surfaces.

Experimental treatment	Clean *n* (%)	Light *n* (%)	Moderate *n* (%)	Heavy *n* (%)
Conditioning film enzymatic solution	4 (44.4)	5 (55.6)	0 (0.0)	0 (0.0)
Enzyme control	0 (0.0)	6 (66.7)	3 (33.3)	0 (0.0)
Detergent component control	4 (44.4)	5 (55.6)	0 (0.0)	0 (0.0)
Powerfoam Plus	7 (77.8)	2 (22.2)	0 (0.0)	0 (0.0)
Hot water	0 (0.0)	6 (66.7)	3 (33.3)	0 (0.0)
Positive control	0 (0.0)	0 (0.0)	2 (22.2)	7 (77.8)
Negative control	9 (100.0)	0 (0.0)	0 (0.0)	0 (0.0)

Protein contamination was assessed categorically using Surface Protein Plus swabs (Neogen, Lansing, Michigan, United States). Comparison of surface cleanliness frequency following experimental treatment with Chi-square analysis (*p* < 0.001).

The results of ATP swabbing assays broadly supported the findings of the protein swabbing assays (Table 5). The CFES demonstrated a marked reduction of ATP (measured in RLU) on both SS (101, *p* < 0.0001) and TPU (112, *p* < 0.0001) when compared to the untreated control positive coupons (SS: 2033, TPU: 1146). Significant reductions were also achieved on SS by enzyme control (566) (209), Powerfoam (267), and hot water (562) (*p* < 0.0001). On TPU coupons, there was less reduction when employing the hot water treatment (624, *p* = 0.007) and Powerfoam Plus (509, *p* = 0.001) when compared to enzyme control (337) and detergent component control (295) (*p* < 0.0001). However, overall, all treatments were able to significantly reduce organic contamination when applied at 40 °C (CFES, detergent component control, enzyme control) or 60 °C (hot water, PowerFoam Plus).

**Table 5:** Least means square analysis of relative light units following ATP Clean Trace swabbing of contact surfaces following experimental treatments.

Treatment	Surface type	
	SS	TPU
Conditioning film enzymatic solution	101.0^b/y^	112.00^b/y^
Enzyme control	566.0^b/y^	337.2^b/y^
Detergent component control	208.7^b/y^	295.2^b/y^
PowerFoam Plus	267.4^b/y^	509.0^b/y^
Hot water	561.9^b/y^	623.8^b/y^
Positive control	2032.9^a/y^	1146.3^a/z^
Negative control	31.6^b/y^	20.2^b/y^

Relative light units were read using Neogen Luminometer on stainless steel (SS) and thermoplastic polyurethane (TPU). ^a,b,c^Least means square within columns lacking a common superscript letter differ within columns (*p* < 0.0001). ^y,z^Least means square within columns lacking a common superscript letter differ within columns (*p* < 0.0001).

### 3.5 Scanning electron microscopy

**Figure 3:**
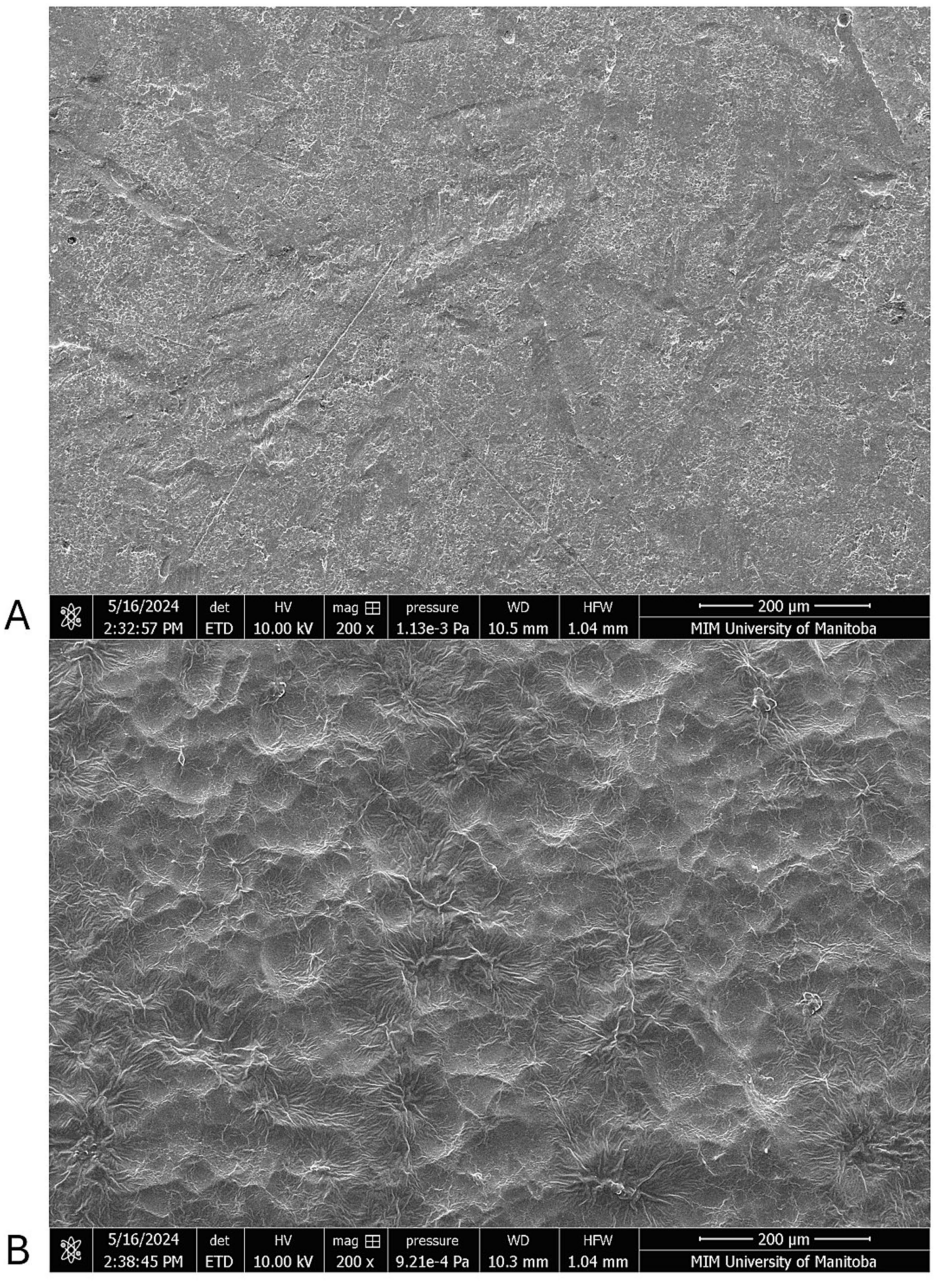
Scanning electron microscopy of stainless steel (SS) and thermoplastic polyurethane (TPU) surfaces under 200X magnification. **(A)** 304-Stainless steel surface. **(B)** Thermoplastic polyurethane surface.

**Figure 4:**
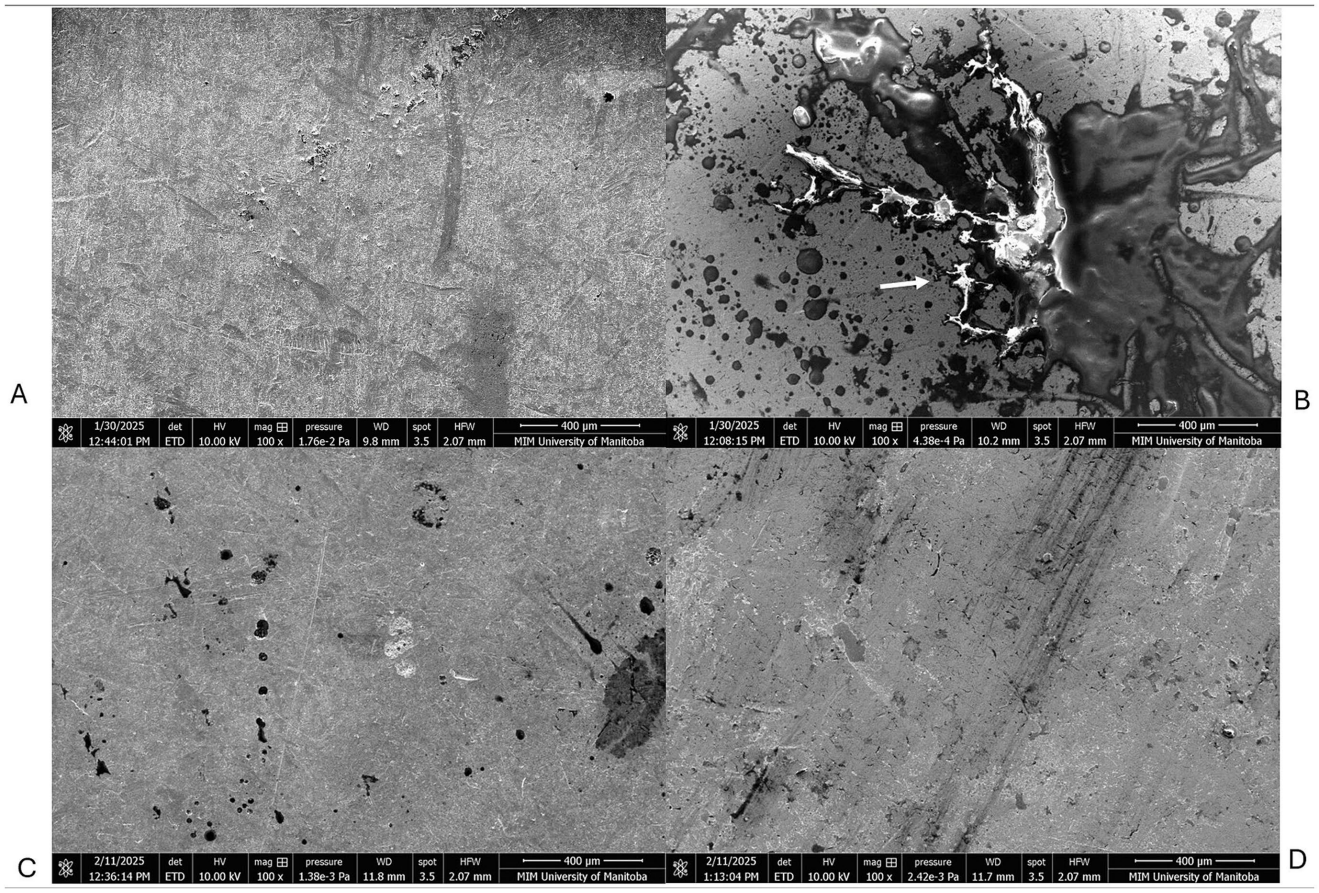
Scanning electron microscopy of stainless-steel contact surfaces. **(A)** Control negative; exposed to distilled water for 16 h at 10 °C. **(B)** Control positive; exposed to beef contamination for 16 h at 10 °C and rinsed with warm water. Arrow indicates an area heavily contaminated by residual organic matter adhered to the surface. **(C)** Contaminated surface treated with CFES. **(D)** Contaminated surface treated with CFES and manually scrubbed with a brush.

**Figure 5:**
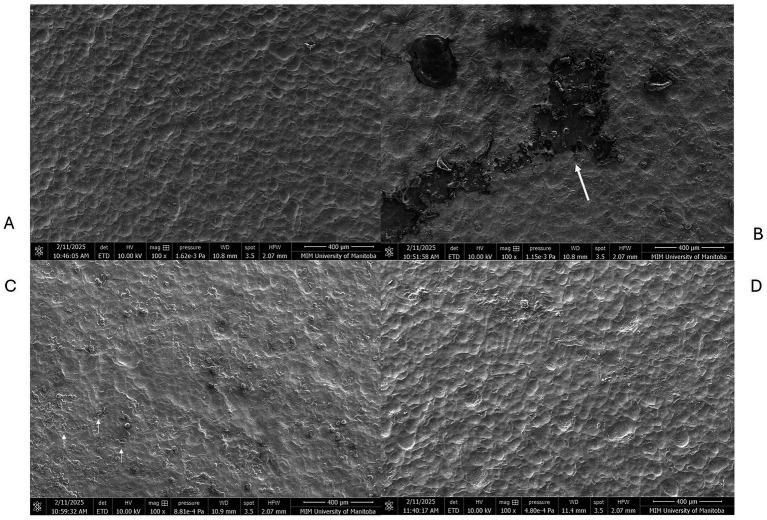
Scanning electron microscopy of thermoplastic polyurethane (TPU) contact surfaces. **(A)** Control negative; exposed to distilled water for 16 h at 10 °C. **(B)** Control positive; exposed to beef contamination for 16 h at 10 °C and rinsed with warm water. Arrow indicates an area heavily contaminated by residual organic matter adhered to the surface. **(C)** Contaminated surface treated with CFES. Arrows indicate beef residues solubilized following treatment, but retained in pitting on TPU surface. **(D)** Contaminated surface treated with CFES and manually scrubbed with brush.

**Figure 6:**
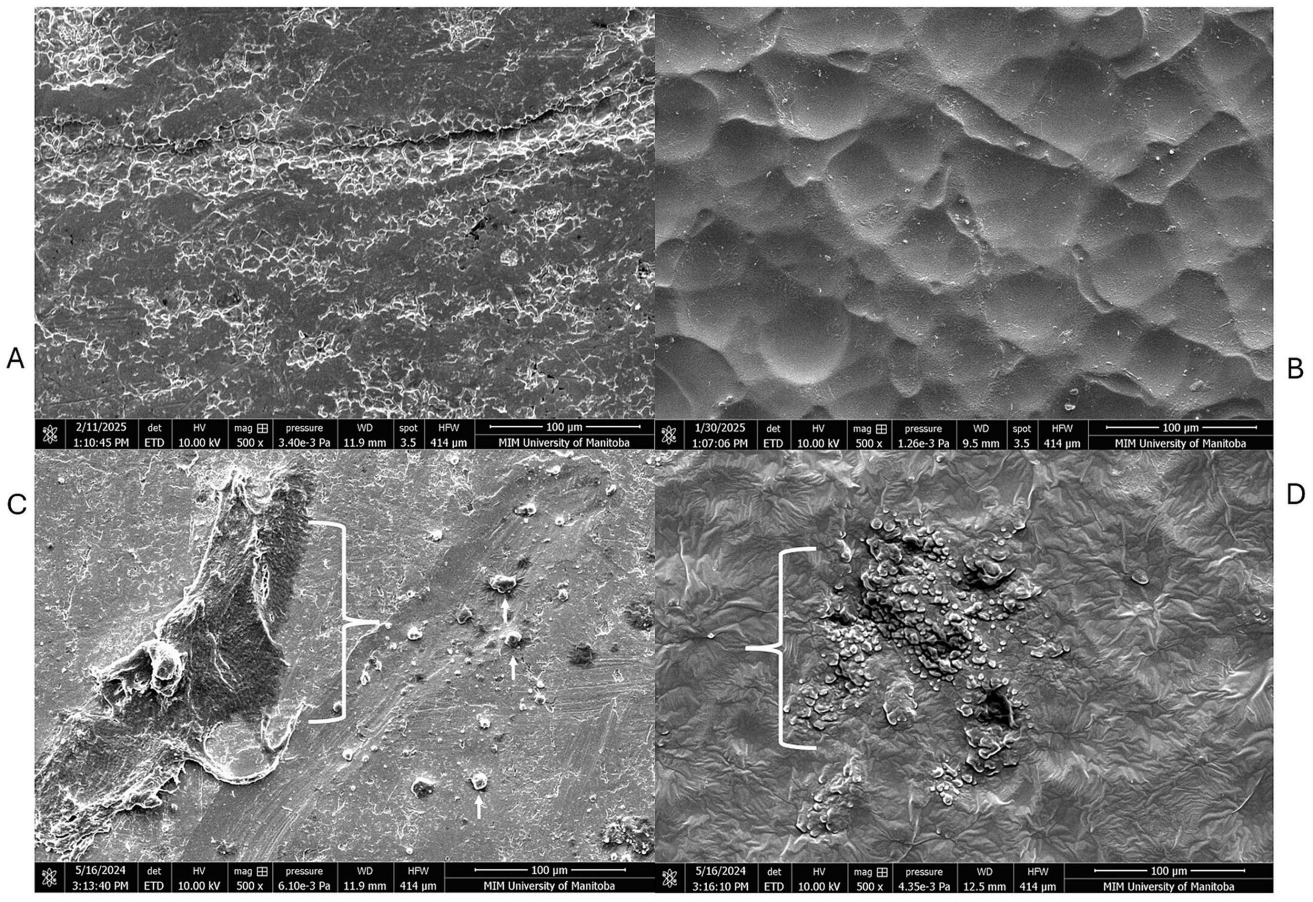
Scanning electron microscopy of thermoplastic polyurethane (TPU) and stainless-steel (SS) surfaces. **(A)** SS control negative; exposed to distilled water **(B)** TPU control negative; exposed to distilled water **(C)** SS surface, contaminated with beef homogenate and washed with 60 °C water. **(D)** TPU surface contaminated with beef homogenate and washed with 60 °C water. The brackets in **(C,D)** indicate areas rich in beef residue retention.

Microscopy using the scanning electron microscope helped to visualize the contamination morphologies on SS and TPU surfaces. The TPU surface exhibited a heterogeneous and irregular topography with pronounced micro-scale features, including ridges and valleys, suggesting greater surface complexity. In contrast, the 304-stainless steel (SS) surface appeared comparatively smoother and more uniform at the same magnification (Figure 3). The FCS treated with the CFES showed less visible debris present on the surface, and inclusion of manual scrubbing with a cleaning brush heightened this reduction, attaining surfaces which closely resemble the control negative panel (Figures 4, 5). Images were also collected which supported our hypothesis that hot water rinsing promotes formation of conditioning films (Figure 6). Both TPU and SS surfaces treated only with hot water (60 °C) showed deposits of organic material after thorough rinsing.

### 3.6 Biofilm disruption

Preliminary testing showed that the CFES had potential to treat bacterial biofilms but was altered in successive testing to improve efficacy (Supplementary Figure 1). In the initial trials, hot water showed the least effective reduction (OD = 0.29, *p* < 0.0001), whereas the PowerFoam Plus, CFES, and the enzyme control solution showed similar levels of reduction (OD = 0.25). The highest reduction was demonstrated by the detergent component control in the preliminary testing (OD = 0.23). Follow-up testing was conducted in which the new enzyme cocktail (*Streptomyces griseus*, lipase from *Pseudomonas cepacia*, cellulase from *Aspergillus niger* and *Trichoderma reesei*, and *α*-amylase from *Bacillus* sp.) was applied at two concentrations (0.05% (w/v) and 0.025% (w/v) of each enzyme product) and with the presence of two detergent component mixtures (SLS: pH 7.41; SLS/BOR pH: 8.97). Since the hot water treatment showed the least reduction (OD = 0.29), it was removed as a potential treatment in subsequent testing, while the solutions containing only SLS or SLS/BOR were included as control treatments.

**Figure 7:**
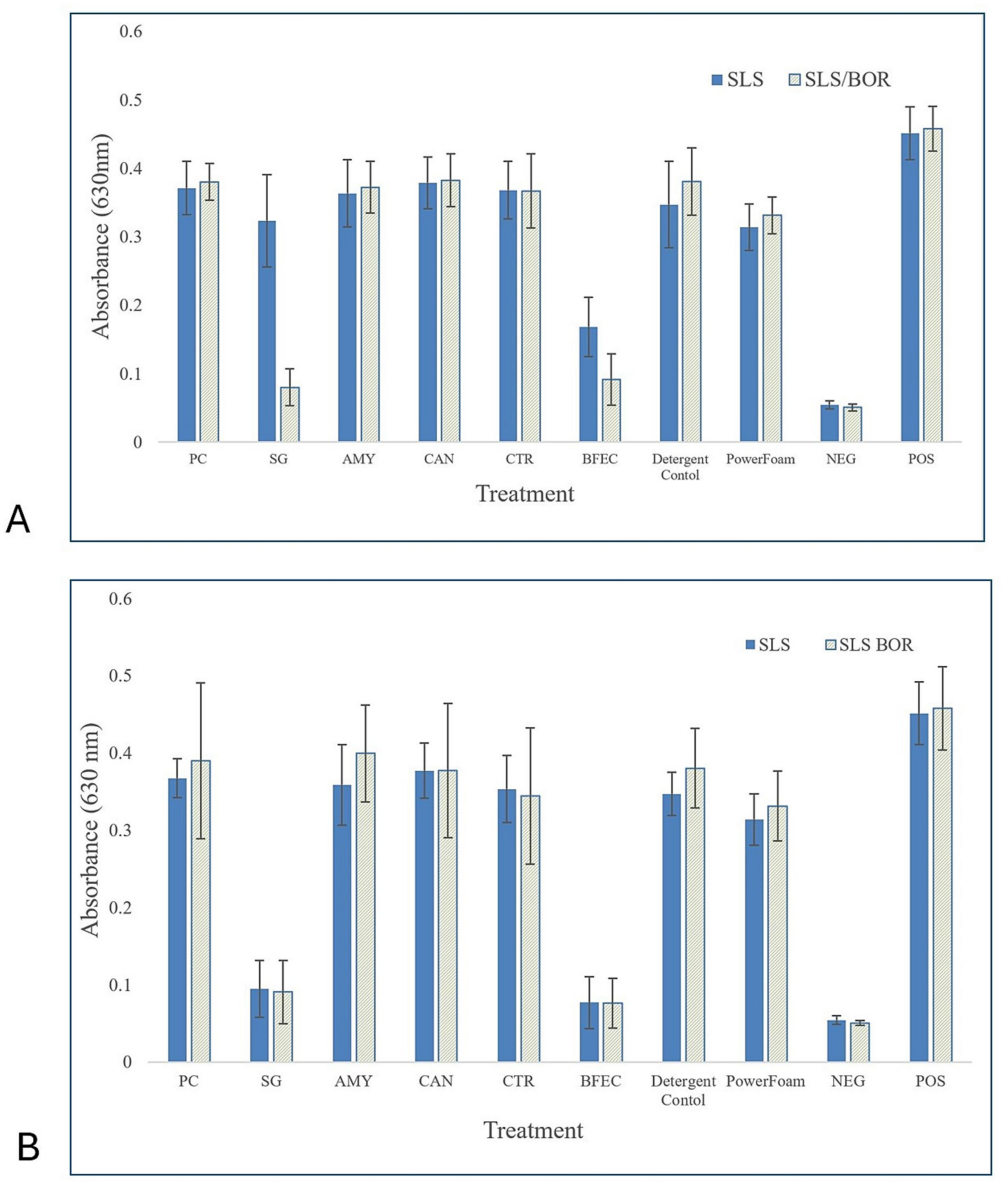
Mean optical density of biofilms following experimental treatments. *Escherichia coli* O157:*H7* R508 single species biofilms were grown in microplates for 6 days at 25 °C before washed thrice with Butterfield’s phosphate buffer and either treated for 10 min with enzyme cocktail, individual enzymes control, or detergent component control (40 °C), or 5 min with PowerFoam plus (60 °C). **(A)** Enzymes applied at high concentration [0.05% (w/v or v/v) and in presence of 0.5% SLS (w/v) or 0.25% SLS (w/v) and 0.25% BOR(w/v)]. **(B)** Enzymes applied at low concentration [0.025% (w/v or v/v) and in presence of 0.5% SLS (w/v) or 0.25% SLS (w/v) and 0.25% BOR(w/v)]. PC, *Pseudomonas cepacia* lipase; SG, *Streptomyces griseus* protease; CTR, cellulase from *Trichoderma reesei*; CAN, cellulase from *Aspergillus niger*; AMY, *α*-amylase from *Bacillus* sp.; SLS, sodium lauryl sulfate; BOR, sodium borate.

The biofilm enzyme cocktail (BFEC) was highly effective in reducing the biofilm extracellular polymeric substance (EPS) (Figure 7). The enzymatic cocktail was able to significantly reduce the biofilm matrix under each set of solution parameters (*p* < 0.0001). Furthermore, it was able to reduce the biofilm matrix to the extent that there was no statistical difference between the enzymatic cocktail and negative control wells when applied at high concentration with SLS/BOR (OD = 0.09, *p* = 0.221), and low concentration with SLS (OD = 0.08, *p* = 0.497) and SLS/BOR (OD = 0.08, *p* = 0.440) (Table 6). Notably, the protease from *Streptomyces griseus*, included in the enzymatic cocktail, showed high efficacy under all conditions when applied in solution on its own, but especially when applied at a higher concentration in a solution with SLS and BOR (OD = 0.08). At a lower concentration [0.025% (w/v)], it also showed significant reduction (*p* < 0.0001) of the EPS matrix, reaching values approaching those of the negative control wells, in both SLS-based solutions (OD = 0.10, *p* = 0.230), and SLS/BOR-based solutions (OD = 0.09, *p* = 0.231).

**Table 6:** Mean biofilm mass following treatment with enzymatic treatments or PowerFoam Plus.

Treatment	High concentration [0.05% (w/v or v/v)], SLS	Low concentration [0.025% (w/v or v/v)], SLS	High concentration [0.05% (w/v or v/v)], SLS/BOR	Low concentration [0.025% (w/v or v/v)], SLS/BOR
Enzyme cocktail	0.17	0.07*	0.09*	0.07*
*Streptomyces griseus* protease	0.32	0.10*	0.08*	0.09*
*Pseudomonas cepacia* lipase	0.37	0.36	0.38	0.39
Cellulase from *Aspergillus niger*	0.38	0.36	0.38	0.38
Cellulase from *Trichoderma reesei*	0.37	0.35	0.37	0.35
α-amylase from *Bacillus sp*	0.36	0.36	0.37	0.40
PowerFoam Plus	0.31	-	0.33	-
Control positive	0.45	-	0.46	-
Control negative	0.06	-	0.05	-

*Escherichia coli O157:H7* R508 single species biofilms were grown in microplates for 6 days at 25 °C before washed thrice with Butterfield’s phosphate buffer and either treated for 10 min with enzyme cocktail, individual enzymes control, or detergent component control (40 °C), or 5 min with PowerFoam plus (60 °C) (SE = 0.0237). *No statistical difference from control negative wells. SLS, sodium lauryl sulfate; BOR, sodium borate.

## 4 Discussion

The present study shows the potential of enzymes as an effective and eco-friendly tool to be explored for enhancing hygiene in meat processing plants. Enzymatic solutions show promise for use either in conjunction with conventional sanitation regimes to optimize food safety or as a potential alternative treatment to reduce some of the intensive input demand of existing protocols, such as freshwater, harsh chemicals, or energy resources, promoting a more sustainable sanitation regimen. In the first set of experiments, food contact surfaces (FCS) were experimentally contaminated under conditions that simulated meat processing facilities. The conditioning film enzymatic solution (CFES), containing lipase and proteases, effectively solubilized meat-based residues deposited on both TPU and SS FCS. Results from ATP CleanTrace and ProteinPlus swabbing assays showed a clear trend toward cleaner surfaces after the CFES was applied (Tables 3–5). Across both surface types, there was a marked reduction in surface ATP levels present on the surface following treatment with CFES, decreasing from an average of 1,590 RLU in positive controls to 107 RLU treated with enzyme solution for 5 min at 40 °C, corresponding to approximately 93% reduction (Table 5). Compared with the industry standard PowerFoam Plus, CFES reduced ATP levels by more than 70% (*p* = 0.038). On TPU surfaces, CFES reduced ATP significantly (*p* < 0.0001) (Table 5) and showed a lower frequency of protein contamination when compared to the control positive coupons (Table 3). Similar results were observed for SS surfaces, with ATP (p < 0.0001) and protein contamination both reduced (Tables 4, 5), even when CFES was applied under reduced operating temperatures (40 °C), and without mechanical spraying or scrubbing. When comparing the two different surface types, higher RLU were detected on the TPU surfaces post-treatment. TPU is a polymeric material composed of soft and hard segments, typically containing urethane linkages, which confer a degree of hydrophobicity and flexibility. According to manufacturer specifications (NuTech Conveyor Components, 2025) the TPU conveyor material used in this study is food-grade, oil- and grease-resistant, and designed for easy cleaning. Previous research has identified TPU conveyor belts as an important reservoir for organic contamination and bacterial persistence in meat processing plants due to their porosity, susceptibility to cracking and roughness, and moist operating conditions (De Medici et al., 2024). Our experiments identified a higher retention of organic material on the TPU surfaces, even when using brand-new conveyor belt material, which may only be exacerbated by repeated use in processing plants. This observation aligns with previous studies demonstrating that STEC biofilms readily form and exhibit increased resilience on TPU materials (Nan et al., 2022). Over time, conveyor belts may develop surface roughness and pitting, creating niches that promote microbial attachment and persistence, thereby increasing contamination risk. Stainless steel is a metallic alloy primarily composed of iron, chromium, and nickel, characterized by a passive chromium oxide layer that provides corrosion resistance and a relatively inert, hydrophilic surface. These chemical differences are expected to influence surface charge and bacterial interactions. Under typical environmental and food-processing pH conditions, stainless steel surfaces generally exhibit a negative surface charge, largely due to the oxide layer (Hedberg et al., 2014). Retention of organic contamination on SS contact surfaces following sanitation treatments has been well evidenced by Evans et al. (2021) and Whitehead et al. (2015). In our experiments, the SS positive control coupons had a high initial level of RLU (SS: 2033, TPU: 1146) (Table 5), which was likely influenced by the hydrophilic properties of the SS promoting retention compared to the hydrophobic properties of the TPU. However, following treatment with the enzymatic treatment or the PowerFoam Plus, the RLU values were lower for SS surfaces, suggesting that be physicochemical properties may be more responsive to cleaning treatments.

The protein detection swabbing assay results generally aligned with the trends seen in the ATP assays (Tables 3–5). Overall, experimental treatments reduced the frequency of detection from predominantly high levels of protein contamination to lower levels. Interestingly, Powerfoam Plus increased the frequency of surfaces classified as clean, despite showing slightly higher ATP detection compared to the enzymatic treatment. The CFES, while able to effectively reduce the ATP levels, still demonstrated some degree of protein. The inconsistencies between the results from the ATP swabs and the protein swabs might be due to the presence of fatty deposits, which are not represented in the protein swabs but can be detected with the ATP luminometer. Moreover, the protein swabs, while helpful in providing a general indication of protein contamination, are less definitive than a quantitative test and are limited in showing discrete differences between treatments.

The findings of the swabbing assays were supported by microscope imaging (Figures 4, 5). On TPU and SS coupons treated with the CFES, there was a clear reduction of organic debris. When surfaces were additionally scrubbed with a brush, as is recommended for optimal sanitation in the food production industry (Marriott et al., 2018), the FCS coupons closely resembled the negative control coupon (exposed only to distilled water), which strengthens the notion that our conditioning film enzymatic solution was effective in promoting thorough removal of organic conditioning films from surfaces, assisted by the addition of emulsifying agents like sodium lauryl sulphate and sodium borate. Our microscopy work largely aligns with similar studies, which use enzymes to target various food-based residues. Using epifluorescence microscopy, Guerrero-Navarro et al. (2022) found that a protease-amylase solution was able to reduce milk-based residues by approximately 85–95%. Boyce et al. (2010) also found enzymatic treatments to diminish milk-based deposits on stainless steel surface panels when employing a proteolytic approach. To our knowledge, our study is the first to microscopically document meat-based residues on FCS prior to- and post-treatment with enzymatic solutions. Although SEM provided qualitative insights into surface morphology, no quantitative measurements of surface roughness or surface charge were performed (Khorasani et al., 2006). Therefore, direct correlations between surface physicochemical properties and microbial behavior cannot be conclusively established. This represents a limitation of the study. Future work should incorporate quantitative surface characterization techniques, including profilometry and surface charge analysis, to better elucidate the relationship between material properties and biofilm formation.

In the second series of experiments, we adjusted the enzymatic solution to target the bacterial biofilm matrix. Three additional enzymes were incorporated into a biofilm enzymatic cocktail (BFEC) specifically tailored for the extracellular polymeric substance. Past studies have used a cocktail-based approach, to provide a variety of enzymes to target substrates present in food residues, and their methods broadly informed our approach (Mnif et al., 2020). The EPS matrix is compositionally complex and varies between bacterial strains and communities, but is largely comprised of proteins, lipids, extracellular nucleic acids, and polysaccharides (Liu et al., 2023). Approaches to biofilm disruption employ enzymes targeting these components to strategically break up the biofilm matrix, typically including various proteases, cellulases, amylases, DNases, and lipases. The present study incorporated a mix of proteases from *Streptomyces griseus*, lipases from *Pseudomonas cepacia*, cellulases from *Trichoderma reesei* and *Aspergillus niger,* and *α*-amylase from *Bacillus* in a solution that was highly effective in reducing EPS matrix secreted by a pathogenic strain of *E. coli* (Figure 7). The application of the enzyme cocktail, particularly when applied at a lower concentration (0.025% (w/v) of each enzyme) and in the presence of 0.25% (v/v) SLS and 0.25% (w/v) BOR showed near eradication of the biofilm matrix. The optical density, proportionate to the biofilm matrix retained after treatment, decreased from OD = 0.46 (control positive) to OD = 0.07 when treated with the BFEC, representing an 83% reduction in biofilm matrix. There was no statistical difference between the negative control wells and the wells treated with the biofilm enzymatic cocktail (*p* = 0.31) (Table 6). Although the BFEC was significantly effective across all application conditions, there was a statistical interaction indicating that the preparation of the enzyme cocktail with 0.25% SLS/0.25% BOR, compared to 0.5% SLS was critical for optimizing the efficacy of treatment. We believe this was due to the adjustment of the pH toward a more alkaline solution with the addition of sodium borate, which may increase the activity of some enzymes present in the solution. Although we aimed to maintain our enzymatic treatments within a neutral pH, the addition of sodium borate shifted the pH from roughly 7.4 to 9.0. While this is a departure from neutral pH, when compared to the 2.5% Powerfoam Plus (pH = 12.6), the solution was much milder. When comparing the efficacy of PowerFoam Plus to our BFEC, our enzyme treatment was more effective under all conditions (*p* < 0.0001) (Figure 7).

Enzyme treatments to degrade biofilm EPS have been extensively studied, and their success is well documented (Kim et al., 2019; Lim et al., 2019; Tsiaprazi-Stamou et al., 2019; Mazaheri et al., 2020; Mnif et al., 2020; Nahar et al., 2021). In general, these experiments found that application of enzyme treatments was successful in targeting the biofilm matrix of various microbial species. In addition to efficacy, these studies also documented several other benefits, such as reduced environmental impact, and more efficient cleaning regimes. Boyce et al. (2010) found that application of enzymatic cleaning solutions was an acceptable alternative to conventional caustic-based procedures. The enzymatic procedure was able to perform effectively at 40 °C and 50 °C and was similar in cost compared to traditional sodium hydroxide and detergent products. A follow-up study employing crude enzymes produced by fungi showed similar effectiveness as reduced temperatures, providing the same environmental benefits (Boyce and Walsh, 2012). Other works by Delhalle et al. (2020) and Guerrero-Navarro et al. (2018) have shown effective enzymatic cleaning strategies for reducing biofilms or organic contamination while operating at lower temperatures. Guerrero-Navarro et al. (2018) reported a 33% reduction in water usage, 29% reduction in temperature, and a 33% reduction in cleaning time upon introduction of an enzymatic cleaning solution. While the present study suggests potential environmental benefits are attainable with the implementation of enzymatic cleaning formulations into meat processing facilities, further research is necessary to optimize their application on an industrial scale.

Despite their promised benefits, the potential of enzymes has only been explored recently in food processing plants. With a rising demand for eco-friendly technologies, enzymes possess great potential for integration into sanitation strategies. However, successful incorporation into food processing plants must appreciate and reflect the diversity of processing techniques and equipment employed across food product industries. Across manufacturing plants, a variety of different sanitation systems are used including clean-in-place (CIP), clean-out-of-place (COP), and open-plant cleaning (OPC) (Timmerman et al., 2016). CIP equipment is commonly used in industries where fluidic food products are circulated, such as milk processing or brining solution pumps (Marriott et al., 2018). COP equipment requires the disassembly of larger equipment structures and is commonly found in food preparation or food service facilities. OPC systems are commonly found in meat fabrication plants. Their design allows for minimal disassembly and reassembly time, and large areas of processing line can be cleaned rapidly while limiting employee labor inputs. OPC systems innately include higher variability regarding temperature, volume of formulation applied, environmental humidity, and contact time. While our enzymatic formulation displayed efficacy when applied in solution to TPU and SS coupons, the experiment was performed under laboratory conditions, which are more closely controlled compared to those employed on a processing plant scale. Furthermore, the use of industry-relevant surfaces, residues, and ATP/protein swabbing, readily available, non-complex methods, compared with complex chemical analyses, which are often not available to industry, supports the applicability of these findings to the meat industry and reinforces their relevance for environmental monitoring practices in meat processing plants.

Therefore, the application of enzymatic solutions will need to be explored in a pilot-scale study to verify scalability and effectiveness. Additionally, while the BFEC was effective in reducing the biofilm matrix secreted by *E. coli* O157:H7, biofilm communities in meat processing plants included a multitude of species, and future work should consider expanding testing to multispecies biofilms. Our study highlights the potential of enzymatic formulations to reduce organic load accumulation during production and to enhance biofilm removal during both operational and post-operational cleaning.

In conclusion, a novel enzymatic formulation consisting of lipases from *Aspergillus oryzae* and *Pseudomonas cepacia*, proteases from *Streptomyces griseus* and papain, a surfactant, (sodium lauryl sulfate) and a detergent builder (sodium borate) improved hygienic properties of SS and TPU surfaces experimentally contaminated with beef while operating at a reduced temperature (40 °C), neutral pH, and mild wastewater profile. An enzymatic cocktail (protease from *Streptomyces griseus*, lipase from *Pseudomonas cepacia*, cellulase from *Aspergillus niger* and *Trichoderma reesei*, and *α*-amylase from *Bacillus* sp.) targeted the biofilm EPS of *E. coli* O157:H7 biofilms and was able to achieve an 83% reduction of the biofilm matrix. Future research should assess the efficacy of enzymatic formulation on a pilot-plant scale and explore ways to optimize the potential of enzymatic sanitation strategies for meat processing plants. In addition, studies should determine the most effective stage for its implementation, whether during operational cleaning or as part of pre-operational sanitation, to maximize its efficiency and practical integration into existing cleaning programs. Enzymatic sanitation solutions represent an eco-friendly and strategic method to improve sanitation and sustainability in the meat processing industry.

## Data Availability

The raw data supporting the conclusions of this article will be made available by the authors, without undue reservation.
