# Supplementary figures and images for: Enhancing sanitation efficiency in red meat processing: a novel enzymatic approach
Source: Front Microbiol. 2026 Apr 21;17:1818470. doi: 10.3389/fmicb.2026.1818470 (PMC13139102; doi:10.3389/fmicb.2026.1818470)

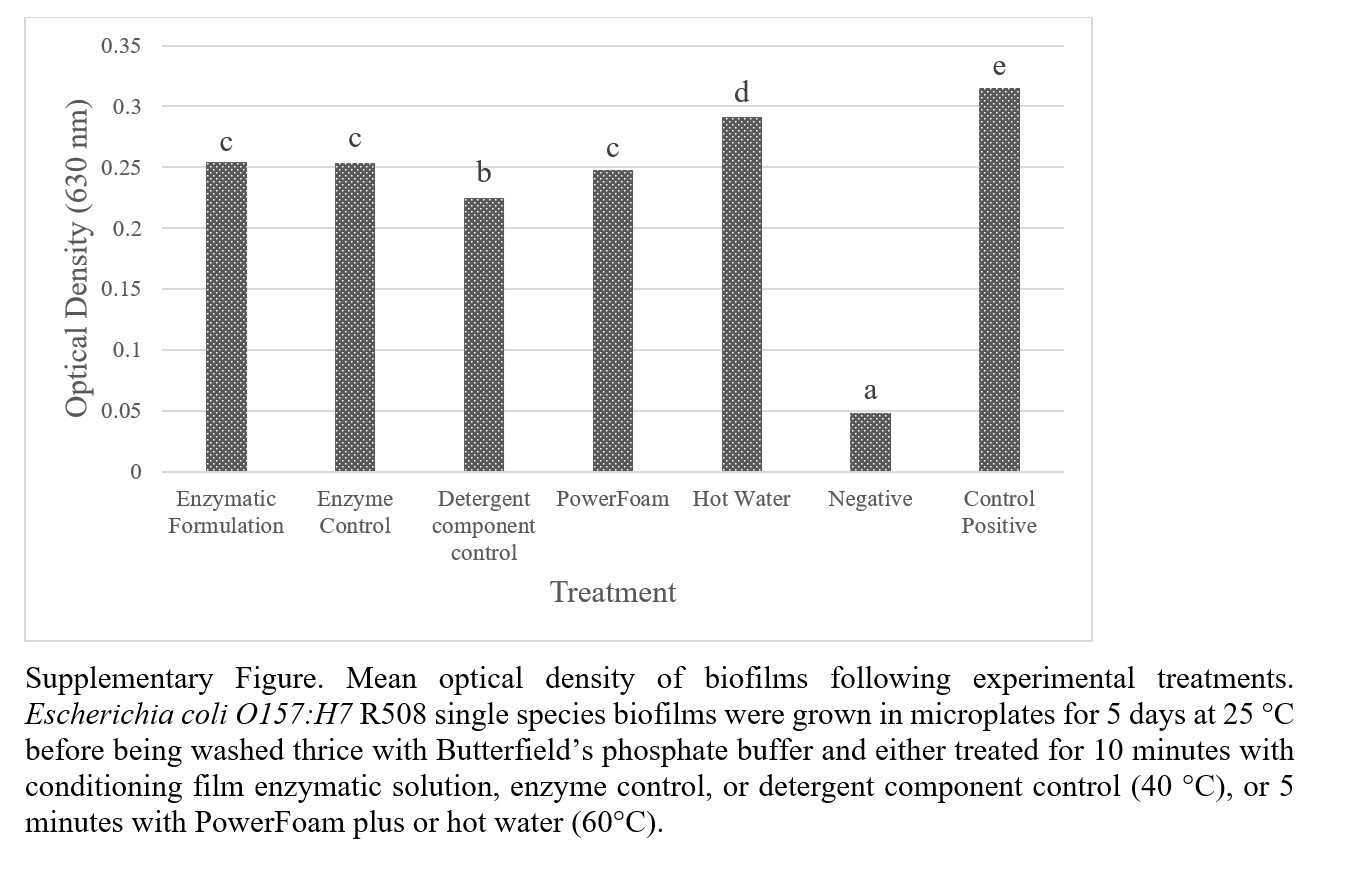

Supplement: Supplementary file 1 [file Image_1.jpeg]

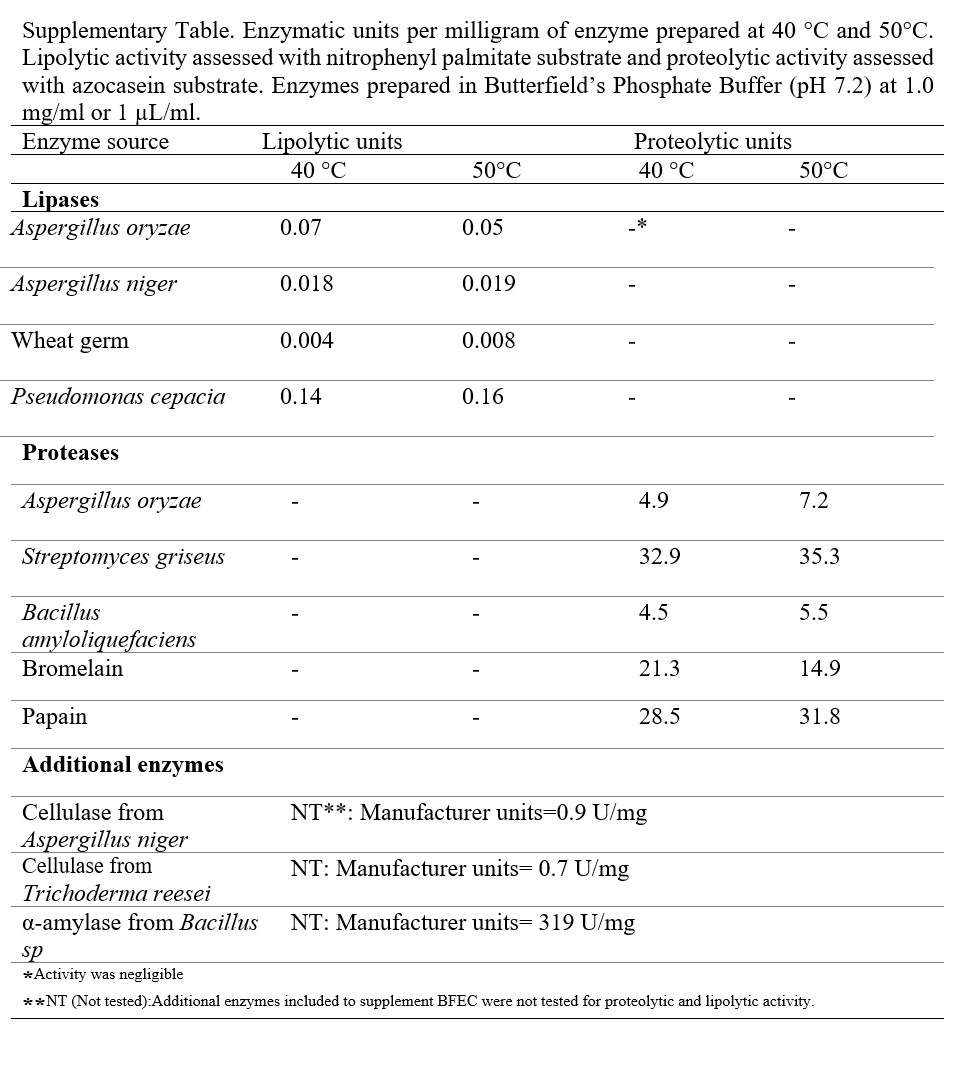

Supplement: Supplementary file 2 [file Image_2.jpeg]
